# Assessing the structure and temporal dynamics of seabird communities: the challenge of capturing marine ecosystem complexity

**DOI:** 10.1111/1365-2656.12434

**Published:** 2015-10-06

**Authors:** Rocío Moreno, Gabriele Stowasser, Rona A. R. McGill, Stuart Bearhop, Richard A. Phillips

**Affiliations:** ^1^ British Antarctic SurveyNatural Environment Research CouncilHigh CrossMadingley RoadCambridgeCB3 0ETUK; ^2^ Scottish Universities Environmental Research Centre (SUERC)Rankine AvenueScottish Enterprise Technology ParkEast KilbrideG75 0QFUK; ^3^ Centre for Ecology and ConservationUniversity of ExeterTremoughPenrynCornwallTR10 9EZUK

**Keywords:** community, diet, pellets, procellariiform, regurgitations, resource partitioning, seabirds, stable isotopes, stomach contents, trophic guilds

## Abstract

1. Understanding interspecific interactions, and the influences of anthropogenic disturbance and environmental change on communities, are key challenges in ecology. Despite the pressing need to understand these fundamental drivers of community structure and dynamics, only 17% of ecological studies conducted over the past three decades have been at the community level.
2. Here, we assess the trophic structure of the procellariiform community breeding at South Georgia, to identify the factors that determine foraging niches and possible temporal changes. We collected conventional diet data from 13 sympatric species between 1974 and 2002, and quantified intra‐ and inter‐guild, and annual variation in diet between and within foraging habits. In addition, we tested the reliability of stable isotope analysis (SIA) of seabird feathers collected over a 13‐year period, in relation to those of their potential prey, as a tool to assess community structure when diets are diverse and there is high spatial heterogeneity in environmental baselines.
3. Our results using conventional diet data identified a four‐guild community structure, distinguishing species that mainly feed on crustaceans; large fish and squid; a mixture of crustaceans, small fish and squid; or carrion. In total, Antarctic krill *Euphausia superba* represented 32%, and 14 other species a further 46% of the combined diet of all 13 predators, underlining the reliance of this community on relatively few types of prey. Annual variation in trophic segregation depended on relative prey availability; however, our data did not provide evidence of changes in guild structure associated with a suggested decline in Antarctic krill abundance over the past 40 years.
4. Reflecting the differences in δ^15^ N of potential prey (crustaceans vs. squid vs. fish and carrion), analysis of δ^15^ N in chick feathers identified a three‐guild community structure that was constant over a 13‐year period, but lacked the trophic cluster representing giant petrels which was identified using conventional diet data.
5. Our study is the first in recent decades to examine dietary changes in seabird communities over time. Conventional dietary analysis provided better resolution of community structure than SIA. However, δ^15^ N in chick feathers, which reflected trophic (level) specialization, was nevertheless an effective and less time‐consuming means of monitoring temporal changes.

## Introduction

Anthropogenic factors, including climate change and overfishing, are among the dominant forces that impact on the structure and dynamics of marine ecosystems (Halpern *et al*. 2008; Hoegh‐Guldberg & Bruno 2010). Oceanographic changes alter energy flow within food webs by increasing or decreasing the amount of primary and secondary production available to consumers (Beaugrand, Luczak & Edwards 2009; Brown *et al*. 2010). In addition, fishing redirects energy flow from pathways involving heavily harvested species to those involving species that are exploited little or not at all (Link & Garrison 2002; Smith *et al*. 2011). Consequently, climate‐ and fisheries‐induced changes in prey availability influence intra‐ and interspecific competition and resource partitioning of marine predators (Sandvik, Coulson & Saether 2008; Forcada & Trathan 2009; Hill, Phillips & Atkinson 2013). Although our understanding of the impact of anthropogenic exploitation on predator populations has improved (Croxall, Trathan & Murphy 2002; Trathan, Forcada & Murphy 2007; Cury *et al*. 2011; Lauria *et al*. 2013), there is a pressing need to develop more integrated evaluations of ecosystem status. However, only 17% of ecological studies in the past three decades have been at the community level (Carmel *et al*. 2013), suggesting that the characterization of changes in food webs at multiple trophic levels remains challenging.

Extensive research on seabirds has demonstrated not only their vulnerability to environmental perturbations, but also their utility, because they integrate information on multiple taxa, as indicators of changes in the wider ecosystem (Croll *et al*. 2005; Frederiksen *et al*. 2006; Piatt, Sydeman & Browman 2007; Einoder 2009; Cury *et al*. 2011). As environmental variation affects different aspects of their feeding ecology, changes in diet, prey capture rates, chick provisioning and growth, and breeding success may reflect impacts ranging from relatively subtle alterations in behaviour to major repercussions for populations (Votier *et al*. 2008; Grémillet & Charmantier 2010; Lewison *et al*. 2012) and can highlight ecosystem‐wide events (Miller & Sydeman 2004; Montevecchi 2007; Moreno *et al*. 2013). Moreover, seabirds have a wide range of ecological roles from secondary to apex consumers, and as scavengers, and whole communities are accessible for sampling during the breeding season; hence, the opportunity exists for developing a reliable, multispecies and multi‐trophic level indicator of the ecosystem that can be used in management and conservation (Frederiksen *et al*. 2006; Piatt, Sydeman & Browman 2007; Grandgeorge *et al*. 2008; Cury *et al*. 2011).

Traditionally, changes in the diet of seabirds are monitored using stomach contents, pellets or, less commonly, direct observations of prey carried by returning adults, or dropped items collected at breeding colonies. Although these approaches can be biased, the results provide reasonable taxonomic resolution (Karnovsky, Hobson & Iverson 2012) and have been invaluable for examining interspecific dietary segregation (Table 1). However, monitoring the diets of a whole community by such methods is a daunting and time‐consuming task. An alternative is to use nitrogen and carbon stable isotope analysis (SIA) of bird tissues, which are less biased but provide coarser taxonomic information and are reliant on a number of assumptions (Layman *et al*. 2012). It is essential, however, to recognize that marine environments usually show complex spatial and temporal variation in baseline isotope signatures due to oceanographic processes (Graham *et al*. 2010). In the Southern Ocean, for example, SIA has been used successfully to describe seasonal changes in the isotopic niche space of the seabird community at South Georgia (Bodey *et al*. 2014). Nevertheless, baseline δ^15^N and δ^13^C change with latitude, sea surface temperature, nutrient and Chl‐a concentration, which is reflected in consumer tissues (Cherel *et al*. 2007; Phillips *et al*. 2009; Stowasser *et al*. 2012), and may obscure feeding relationships and prevent the estimation of trophic level or specific prey consumption (Ménard *et al*. 2007; Moreno *et al*. 2011; Roscales *et al*. 2011). Therefore, any study of a seabird community should consider the complications associated with high variability in foraging ranges and use of water masses with potentially differing isotopic baselines.

**Table 1:** Sources of diet information used in this study

Species	Type of diet samples	Season	Month	Age	Source
Antarctic prion	Regurgitations	1974	February–March	Adults	Prince (1980)
	Regurgitations	1986	February	Adults	Croxall, Prince & Reid (1997)
	Regurgitations	1991–1992	February	Adults	Liddle (1994)
	Regurgitations	1994	February	Adults	Reid, Croxall & Edwards (1997a)
Black‐browed albatross	Regurgitations	1975–1976	February–March	Adults	Prince (1979)
	Regurgitations	1986	February	Adults	Croxall, Prince & Reid (1997)
	Regurgitations	1994	February	Adults	Croxall, Reid & Prince (1999)
	Regurgitations	1996–2000	February–May	Chicks	Xavier, Croxall & Reid (2003)
Blue petrel	Regurgitations	1974	December–January	Adults	Prince (1980)
Common diving petrel	Stomach contents	1973–1974	December–March	Chicks	Payne & Prince (1979)
	Stomach contents	1987	November–February	Adults	Reid *et al*. (1997b)
Fairy prion	Regurgitations	1983	December–February	Adults	Prince & Copestake (1990)
Grey‐headed albatross	Regurgitations	1975–1976	February–March	Adults	Prince (1980)
	Regurgitations	1986	February	Adults	Croxall, Prince & Reid (1997)
	Regurgitations	1994	February	Adults	Croxall, Reid & Prince (1999)
	Regurgitations	1996–2000	February–May	Chicks	Xavier, Croxall & Reid (2003)
Light‐mantled sooty albatross	Regurgitations	1977–1978	November–April	Adults and chicks	Thomas (1981)
Northern giant petrel	Regurgitations	1980–1981	January	Chicks	Hunter (1983)
South Georgia diving petrel	Stomach contents	1972–1973	December–March	Chicks	Payne & Prince (1979)
	Stomach contents	1986–1987	December–March	Adults	Reid *et al*. (1997b)
Southern giant petrel	Regurgitations	1980–1981	January	Chicks	Hunter (1983)
Wandering albatross	Regurgitations	1983–1984	May–September	Chicks	Croxall, North & Prince (1988)
	Regurgitations	1983–1984	May–September	Chicks	Rodhouse, Clarke & Murray (1987)
	Pellets	1999–2000	May–August	Chicks	Xavier, Croxall & Reid (2003)
White‐chinned petrel	Regurgitations and stomach contents	1986	February	Adults	Croxall, Prince & Reid (1997)
	Regurgitations	1996 and 1998	January–March	Adults	Berrow & Croxall (1999)
Wilson's storm petrel	Regurgitations	1985	March	Adults	Croxall, North & Prince (1988)

The breeding season is given as the year in which the chicks fledged, for example austral summer 1985/1986 is denoted 1986 etc.

The Southern Ocean has been influenced not only by sealing, whaling and fishing over the last two centuries (Murphy *et al*. 2007; Trathan & Reid 2009), but also shows some of the strongest signals of global climate warming (Levitus *et al*. 2000; Gille 2002). Retrospective analyses suggest that abundance of Antarctic krill *Euphausia superba* in some regions of the Southern Ocean may have declined in the last 40 years as a consequence of reduced sea‐ice extent and duration (Atkinson *et al*. 2004; but see Loeba & Santorab 2015; Steinberg *et al*. 2015). Thus, establishing feasible methods to describe and monitor the structure and function of Antarctic communities is imperative for a better understanding of ecosystem status, and for developing sustainable management strategies. One of the major breeding sites in the Southern Ocean for seabirds, including many threatened species, is South Georgia (Clarke *et al*. 2012). During the last four decades, the feeding ecology of most species breeding at this site has been characterized using conventional techniques, but until now, there was no attempt to integrate this wealth of dietary information in a quantitative analysis of variation between and within foraging guilds. Nor has there been a formal test of the reliability or limitations of SIA as a tool for quantifying trophic community structure where there is high spatial and temporal heterogeneity in environmental baselines.

Aiming to better understand resource partitioning within the procellariiform community breeding at Bird Island, South Georgia, we reviewed information from conventional dietary assessment of 13 sympatric species between 1974 and 2002 to (i) assess community structure, (ii) examine for the first time, evidence for temporal changes in this structure and (iii) investigate whether the same conclusions would have been drawn if the prey data were resolved only to higher taxonomic levels (i.e. family, rather than genus or species), which would involve much reduced monitoring effort. Furthermore, by comparing conventional dietary information with stable isotope ratios (δ^15^N and δ^13^C) in seabird chick feathers collected over a 13‐year period in relation to those of their potential prey from different water masses (i.e. with wide intraspecific variability in isotopic signatures), we highlight several important issues that were unresolved in the Antarctic and elsewhere.

## Material and methods

### Study area and species

**Figure 1:**
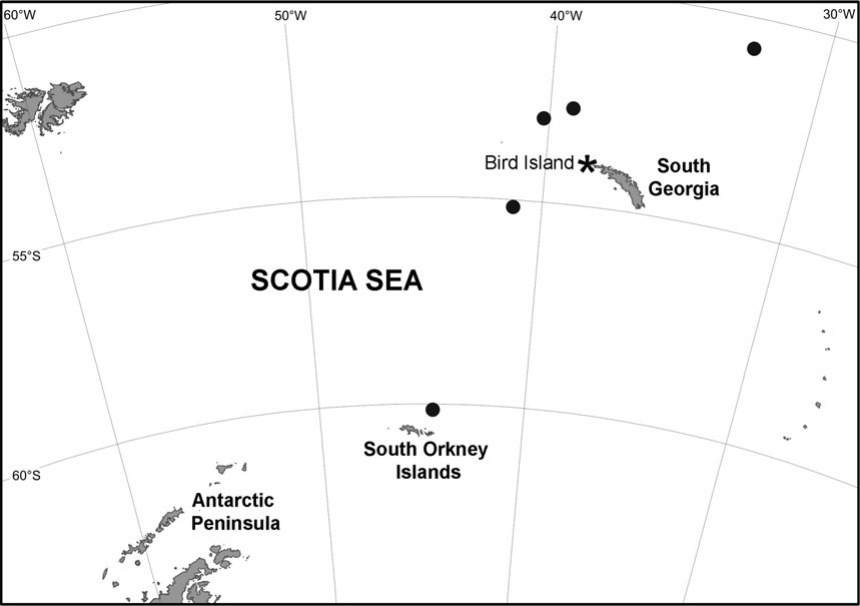
Map of the sampling area. Stations sampled for potential prey within the main foraging distribution of the seabird species from this study are indicated by enlarged circles (Stowasser *et al*. 2012).*is marking Bird Island, the study area. The island where the procellariiform community monitored in this study breeds.

Bird Island (54°00′ S, 38°03′ W) is situated close to the north‐west tip of South Georgia, in the maritime subantarctic (Fig. 1). The waters of the South Georgia shelf and slope are characterized by phytoplankton concentrations and rates of primary production that are among the highest in the Southern Ocean (Atkinson *et al*. 2001), hence the importance of this archipelago for breeding seabirds (Clarke *et al*. 2012). During several austral summers covering a 13‐year period (here and afterwards, the breeding season is given as the year in which the chicks fledged, e.g. austral summer 2001/02 is denoted 2002, etc.), we collected a random sample of body feathers from chicks of 11 sympatric species of Procellariiform (wandering albatross *Diomedea exulans* – 79 individuals in total, black‐browed albatross *Thalassarche melanophris* – 51*,* grey‐headed albatross *T. chrysostoma* – 58, light‐mantled sooty albatross *Phoebetria palpebrata* – 34, northern giant petrel *Macronectes halli* – 59, southern giant petrel *M. giganteus* – 60, white‐chinned petrel *Procellaria aequinoctialis* – 39, blue petrel *Halobaena caerulea* – 19, Antarctic prion *Pachyptila desolata* – 19, South Georgia diving petrel *Pelecanoides georgicus* – 2 and common diving petrel *P. urinatrix* – 6) to analyse δ^15^N and δ^13^C. The influence of spatial variation in prey isotope signatures was examined by comparing stable isotope ratios of chicks with those of 20 species of crustacean, fish, squid and carrion sampled in five locations within the birds' foraging distributions at sea (Fig. 1). These sampling locations reflected a wide spatial range of δ^15^N baselines, from cold, less productive waters in the south of the Scotia Sea, across highly productive waters around South Georgia, to the mixed Antarctic and subantarctic waters of the Polar Frontal Zone, encompassing much of the natural variability in nutrient sources and environmental conditions in the region (Stowasser *et al*. 2012). In addition, we included stable isotope data from six species of squid obtained from diet samples collected from the same procellariiform community at Bird Island (Anderson *et al*. 2009). Together, these prey species represent 73% of items in the diet recorded at the community level.

### Isotopic analyses

Feather samples were washed in chloroform : methanol (2 : 1 v/v) solution, dried, stored in sealed plastic bags and then later ground to a fine powder in a freezer mill operating at liquid nitrogen temperature prior to SIAs. Carbon and nitrogen isotope ratios for feathers were measured by continuous‐flow isotope ratio mass spectrometry (CF‐IRMS) using both a Carlo Erba (model NA 1500) EA linked to a Finnigan Tracer Mat and a Costech (model ECS 4010) EA combined with a Thermo Finnigan Delta Plus XP. Approximately 0·7 mg of each sample was combusted in a tin cup for the simultaneous determination of carbon and nitrogen isotope ratios. Two internal laboratory standards (Sigma‐Aldrich gelatin and LSTD6 – a solution of sucrose and ammonium sulphate, St Louis, MO, USA) were analysed for every 8–10 unknown samples in each analytical sequence with the Carlo Erba system, and three laboratory standards (Sigma‐Aldrich gelatin, and 2 Sigma‐Aldrich alanine solutions one enriched with ^13^C and one with ^15^N) with the Costech system, assuring good matching of results and allowing any instrument drift to be corrected. Stable isotope ratios were expressed in δ notation as parts per thousand (‰) deviation from the international standards V‐Pee Dee Belemnite (carbon) and AIR (nitrogen), according to the following equation:

$$\delta X=\left[\left(\frac{R\;\text{sample}}{R\;\text{standard}}\right)-1\right]\times 1000$$

where *X* is ^15^N or ^13^C, and *R* is the corresponding ratio ^15^N/^14^N or ^13^C/^12^C. Measurement precision of both δ^15^N and δ^13^C was estimated to be ≤0·2‰. All values presented are the mean ± 1standard deviation unless otherwise stated**.**

### Diet data sources

Data on diets from regurgitations, pellets and stomach contents of 13 species (wandering albatross, black‐browed albatross*,* grey‐headed albatross, light‐mantled sooty albatross, northern giant petrel, southern giant petrel, white‐chinned petrel, blue petrel*,* Antarctic prion*,* fairy prion *Pachyptila turtur*, Wilson's storm petrel *Oceanites oceanicus*, South Georgia diving petrel and common diving petrel), all collected at Bird Island, were obtained mainly from published sources and are summarized in Table 1. Prey species were summarized into 120 groups based on identification of the lowest taxonomic level: 24 to genus and 96 to species level. Dietary composition was expressed as percentage wet mass of all ingested prey, either measured or reconstructed (see below), excluding prey that were unidentified, or classified as ‘other'. If diet information from the same samples was described in separate papers (fish and cephalopod prey of wandering albatross from 1983 and 1984) or split into tables or results within the same article (diets of white‐chinned petrels from 1996 and 1998, light‐mantled sooty albatross from 1977 to 1978, diving petrels from 1972 to 1973 and 1973 to 1974, Wilson's storm petrel from 1985, Antarctic prion from 1974, grey‐headed and black‐browed albatross from 1975 to 1976, and northern and southern giant petrel from 1980 and 1981), overall composition was calculated accordingly (for references, see Table 1). If data were expressed in terms of numerical abundance, values were converted to mass by multiplying the number of prey items by the estimated mass (diet of diving petrels from 1986 and 1987 – see Table 1). Sufficient diet data were available for wandering albatross, black‐browed albatross, grey‐headed albatross, white‐chinned petrel, South Georgia diving petrel and Antarctic prion, to enable annual comparisons within and between species at three different taxonomic levels (species, family and group – crustaceans, squid, fish and carrion).

### Statistical analysis

Comparative analyses of diet among years, and between different techniques (SIA and stomach content assessment), were carried out with the PRIMER software package (Plymouth Routines In Multivariate Ecological Research, version 6; Clarke and Gorley, 2006). A variety of resemblance metrics are available, which offer different advantages and disadvantages depending on the context (Somerfield, Clarke & Olsgard 2002; Clarke, Somerfield & Chapman 2006). We calculated Bray–Curtis similarity indices, which are unaffected if taxa are absent for both samples that are being compared. This is because species can be absent for many different reasons, and it is counter‐intuitive to infer that two samples are similar because neither contains particular species (Clarke, Somerfield & Chapman 2006). Diet composition and trophic niche segregation were quantified using hierarchical agglomerate clustering and non‐metric multidimensional scaling (using in both cases, the Bray–Curtis similarity index), followed by analysis of similarities (anosim). The key output of the pairwise tests carried out by anosim is an *R* value that gives an absolute measure of group separation on a scale of 0 (indistinguishable) to 1 (all similarities within groups are less than any similarity between groups). *R* > 0·75 indicates that the groups are well separated, *R* > 0·5 reflects an overlap but a clear difference, and *R* < 0·25 means that the groups are barely separable (Plymouth Routines in Multivariate Ecological Research, version 6; Clarke and Gorley, 2006). We also conducted Bray–Curtis analyses of SI ratios, to enable a comparison between these and the conventional diet data without any potential confounding effect of a difference in methodology.

The proportions of the variance in δ^15^N and δ^13^C explained by prey group (crustaceans, squid, fish and carrion) and prey species (all individual species in each group) were investigated using a combination of random effects models and variance components analysis. The random effects model was fitted using the package *nlme* in the programme r. In the global model, the response variable was δ^15^N or δ^13^C, and the random effects were species nested within prey group, fitted with normal errors and an identity link. Model selection was performed using backward‐stepwise removal of each of the random effects, with the significance of the consequent increase in residual variance tested using anova. Variance components, expressed as proportions of the total variance, were calculated from the selected model using the r package *ape*.

## Results

**Figure 2:**
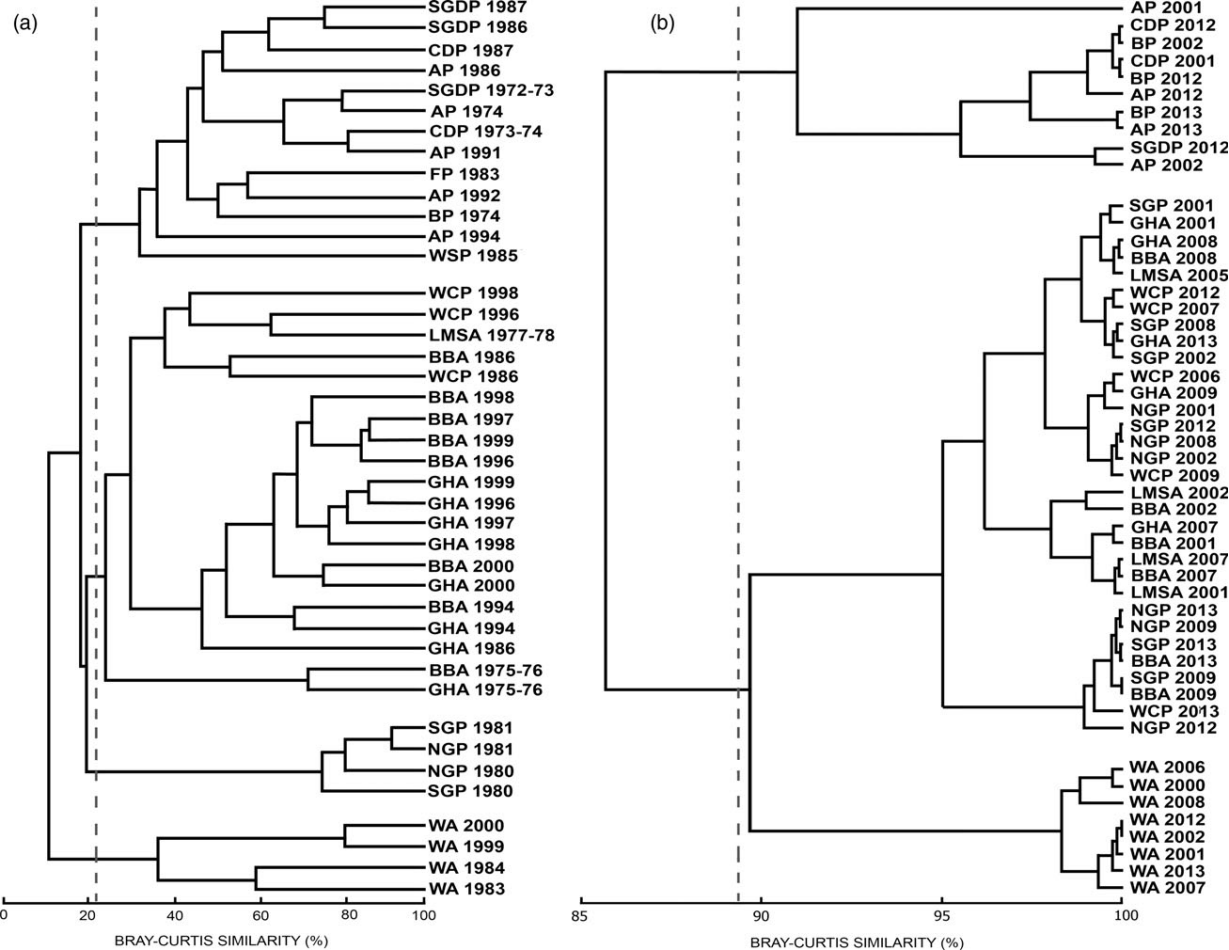
Dendrograms based on (a) contribution by wet mass of different components at species level in conventional diet samples obtained from 13 procellariiform species (WA, wandering albatross; BBA, black‐browed albatross; GHA, grey‐headed albatross; LMSA, light‐mantled sooty albatross; NGP, northern giant petrel; SGP, southern giant petrel; WCP, white‐chinned petrel; BP, blue petrel; AP, Antarctic prion; FP, fairy prion; SGDP, South Georgia diving petrel; CDP, common diving petrel; WSP, Wilson's storm petrel) and (b) δ^15^ N in feathers from chicks of 11 procellariform species sampled at Bird Island, South Georgia. Dashed lines in dendrograms indicate the trophic guilds significant at *P* < 0·05 and defined by anosim analysis.

**Figure 3:**
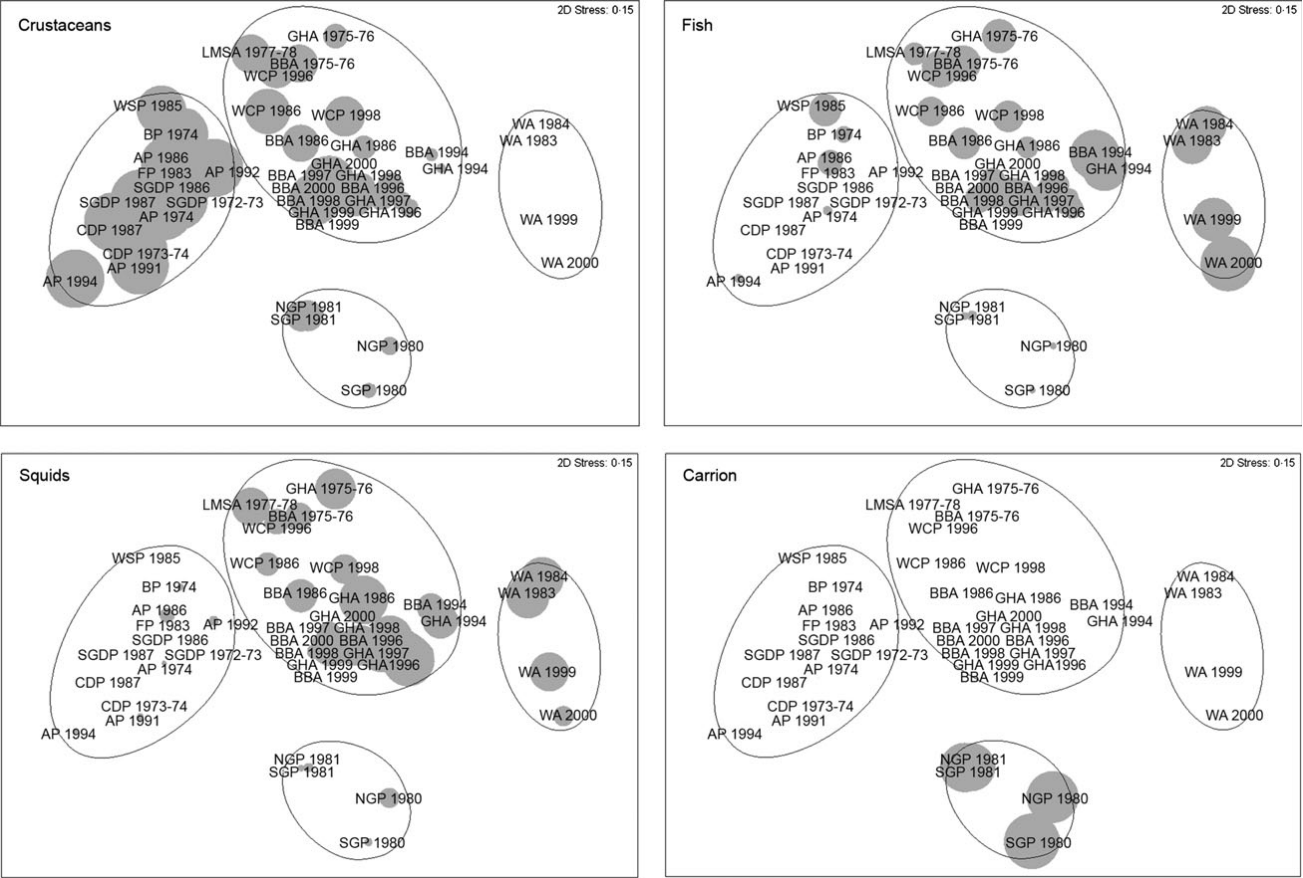
Multidimensional scaling of Bray–Curtis similarities based on contribution by wet mass of different components at species level in conventional diet samples from 13 procellariiform species sampled at Bird Island (WA, wandering albatross; BBA, black‐browed albatross; GHA, grey‐headed albatross; LMSA, light‐mantled sooty albatross; NGP, northern giant petrel; SGP, southern giant petrel; WCP, white‐chinned petrel; BP, blue petrel; AP, Antarctic prion; FP, fairy prion; SGDP, South Georgia diving petrel; CDP, common diving petrel; WSP, Wilson's storm petrel), South Georgia, from several years (Table 1). Superimposed circles of increasing size represent increasing consumption of crustaceans, fish, squid and carrion in the four main species clusters defined by analysis of similarities – anosim (ellipses).

Results of the comparison in conventional diet of 13 species at taxonomic species level are shown in Figs 2a and 3. This generated four significantly different trophic guilds (*R* = 0·78, *P *< 0·001): (i) Antarctic prion, fairy prion, blue petrel, common diving petrel, South Georgia diving petrel and Wilson's storm petrel; (ii) black‐browed albatross, grey‐headed albatross, light‐mantled sooty albatross and white‐chinned petrel; (iii) northern giant petrel and southern giant petrel; and (iv) wandering albatross.

**Figure 4:**
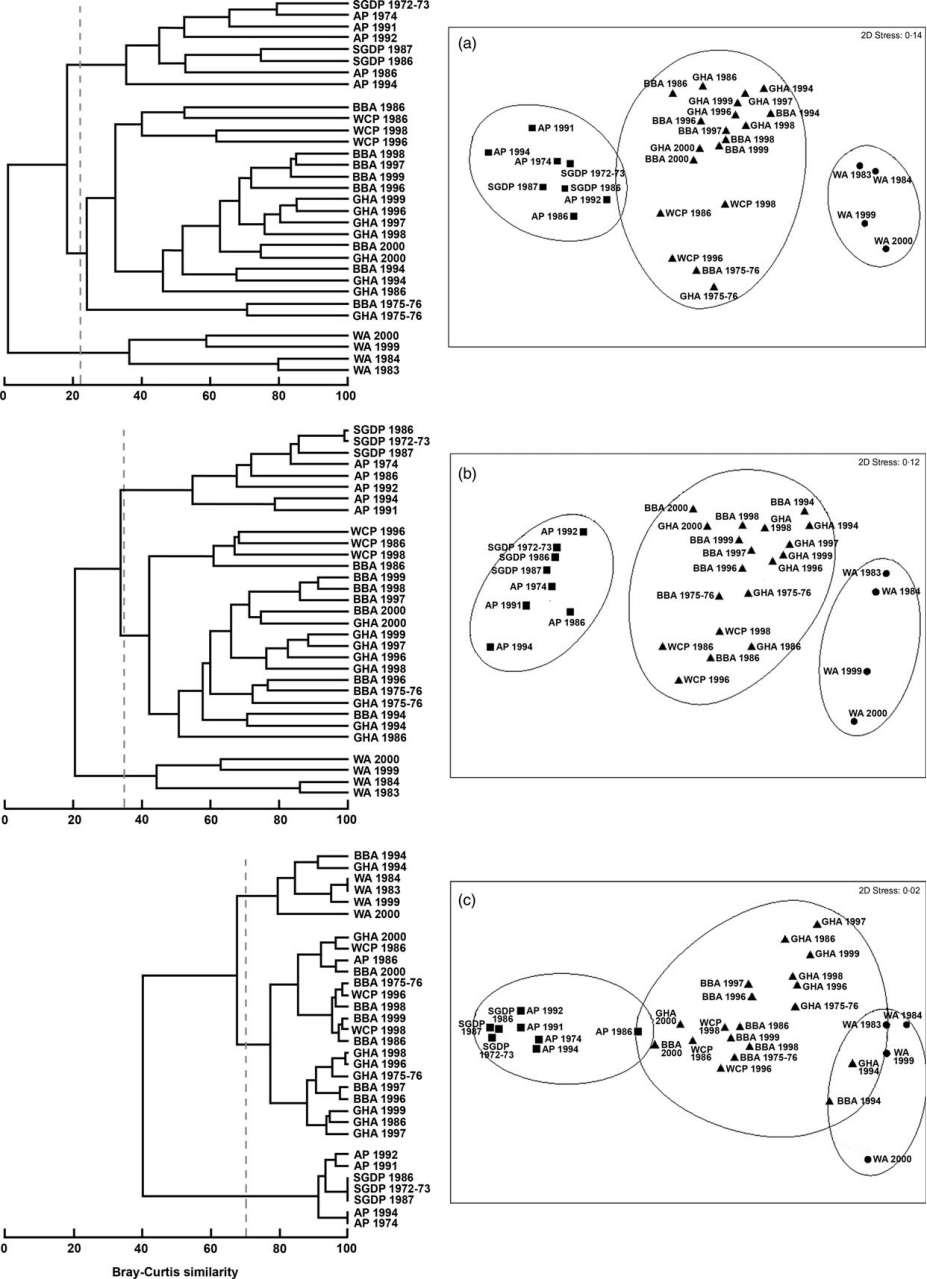
Dendrogram (left) and multidimensional scaling (right) of conventional diet data from six procellariiform species sampled in different years at Bird Island, South Georgia (WA, wandering albatross; BBA, black‐browed albatross; GHA, grey‐headed albatross; WCP, white‐chinned petrel; AP, Antarctic prion; SGDP, South Georgia diving petrel) from several years (Table 1) based on contribution by mass at three different taxonomic levels: (a) species, (b) family and (c) group. Dashed lines in dendrograms at 23%, 35% and 70% diet similarity indicate the three trophic guilds significant at *P* < 0·05 and defined by anosim analysis.

For the six seabird species for which there were 3 or more years of detailed conventional diet data, the species were grouped into the same three significantly different trophic guilds when prey taxonomic resolution was at species (*R* = 0·84, *P* < 0·001) and family level (*R* = 0·88, *P *< 0·001), regardless of year. These guilds comprised the following: (i) wandering albatross; (ii) Antarctic prion and blue petrel; and (iii) black‐browed albatross, grey‐headed albatross and white‐chinned petrel (Fig. 4). In contrast, the species were no longer grouped into the same three trophic guilds when diet data were aggregated at group level. Although anosim analysis showed a pattern of strong niche segregation, diet similarity values were sensitive to the taxonomic level of diet categorization (see similarity values in Fig. 4). For example, in the analysis at prey species and family level for black‐browed and grey‐headed albatrosses, there was a greater similarity in diet between the two species in the same year (i.e. 1994 and 2000), than within each species in different years. However, overlap between the diet of grey‐headed and black‐browed albatrosses in 2000 and white‐chinned petrel and Antarctic prion in 1986, and overlap between the diet of wandering albatrosses in 1983, 1984, 1999 and 2000 and grey‐headed and black‐browed albatrosses in 1994, appeared as an artefact of analysis of diet at the coarsest level (by group).

**Figure 5:**
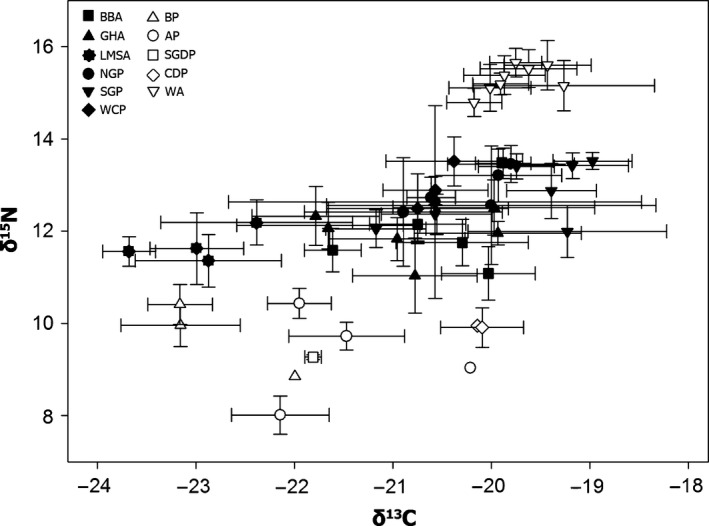
Mean δ^15^ N and δ^13^ C (±SD) in feathers of chicks of 11 procellariiform species (WA, wandering albatross; BBA, black‐browed albatross; GHA, grey‐headed albatross; LMSA, light‐mantled sooty albatross; NGP, northern giant petrel; SGP, southern giant petrel; WCP, white‐chinned petrel; BP, blue petrel; AP, Antarctic prion; SGDP, South Georgia diving petrel; CDP, common diving petrel) sampled at Bird Island, South Georgia, during several years covering a 13‐year period.

**Figure 6:**
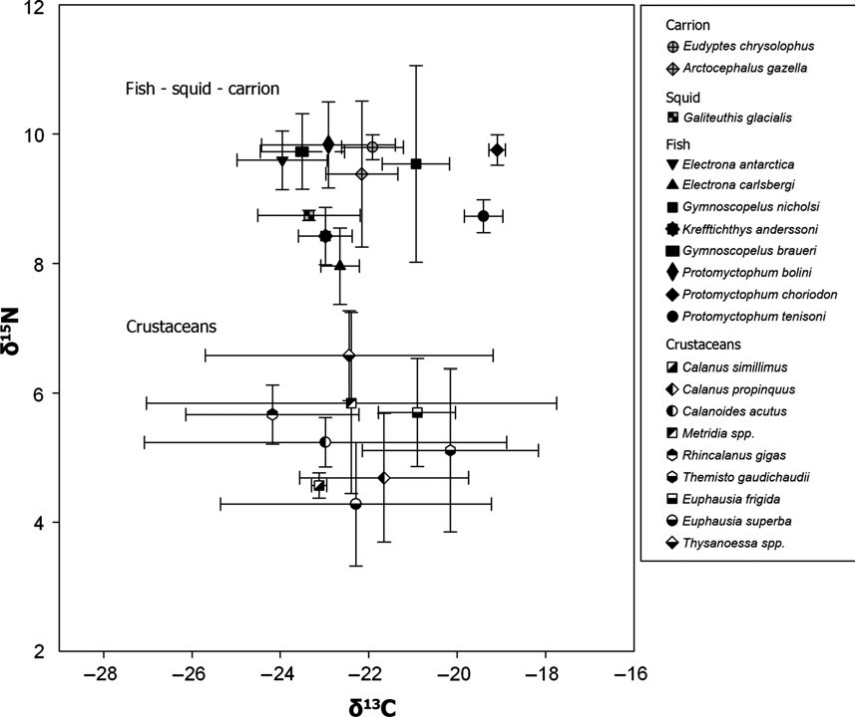
Mean δ^15^ N and δ^13^ C (±SD) in 20 species of crustacean, fish, squid and carrion (fur seals *Arctocephalus gazella* and Macaroni penguin *Eudyptes chrysolophus*) sampled in four locations within the birds' foraging distribution at sea that differed greatly in temperature, productivity and nutrients (Stowasser *et al*. 2012).

There was no significant temporal trend in δ^15^N in chick feathers of the 11 species that were sampled in multiple years. In cluster analyses, these species were grouped into three significantly different trophic guilds (*R* = 0·82, *P *< 0·001): (i) Antarctic prion, blue petrel, common diving petrel and South Georgia diving petrel; (ii) black‐browed albatross, grey‐headed albatross, light‐mantled sooty albatross and white‐chinned petrel, northern giant petrel and southern giant petrel; and (iii) wandering albatross (Fig. 2b). The relative variability between years and among species is clearly illustrated in a standard δ^15^N–δ^13^C biplot (Fig. 5). Note that there were no significant differences in δ^13^C between the main groups of potential prey (Fig. 6), and hence the relationships between δ^13^C and distribution will be explored in more detail in another paper.

In terms of δ^15^N of prey (Fig. 6), removal of the random effects of both the prey group and species terms resulted in significant increases in the residual deviance (prey group; LR = 37·38, d.f. = 1, *P* < 0·0001: species; LR = 110·9, d.f. = 2, *P* < 0·0001). Both terms were therefore retained in the selected model. Variance components analysis of this model revealed that prey group (group 1 = crustaceans; group 2 = carrion, squid and fish) explained 82% of the variation in δ^15^N, species explained 8%, and residual variance was 11%. The variability was over 10 times greater across the prey groups than within individual prey species sampled in areas of different productivity.

In terms of δ^13^C (Fig. 6), the removal of the prey group term resulted in a significant increase in deviance (LR = 28·3, d.f. = 1, *P* < 0·0001), but the removal of species did not (LR = 0·0, d.f. = 1, *P* > 0·9). Variance components were therefore calculated from the model that included prey group but omitted species. Prey group explained only 1·6% of the variance, and the remainder (98·4%) was residual.

## Discussion

Our study highlighted that although the procellariiform seabird community from Bird Island includes small‐to‐large species from different trophic levels and with disparate foraging strategies, only 15 prey species comprised three‐quarters of their diet. The cluster analyses of conventional diet indicated that despite differences in the degree of niche segregation between years depending on availability of prey, there was no evidence of a consistent change in the trophic guild structure related to the suggested decline of krill in the last 40 years (Atkinson *et al*. 2004). Similarly, analysis of SI data from chick feathers did not indicate substantial changes within the 13‐year study period and provided a similar, if somewhat less resolved indication of trophic guild structure to the conventional diet analysis (missing a cluster representing the carrion‐feeding giant petrels). Our detailed picture of a diverse Antarctic seabird community demonstrates that analysis of diet composition at higher taxonomic levels can provide reliable insights into community dynamics. In addition, so long as the potentially confounding influence of a complex underlying marine isoscape can be overcome, SIA is an effective and less time‐consuming means of assessing temporal changes in community trophic structure.

The analysis of conventional diet data collected between 1974 and 2002 for the seabird community breeding at Bird Island highlighted a four‐guild structure defined by species feeding mainly on crustaceans (Antarctic prion, fairy prion, blue petrel, common diving petrel, South Georgia diving petrel, Wilson's storm petrel), large fish and squid (wandering albatross), a mixture of crustaceans, small fish and squid (black‐browed albatross, grey‐headed albatross, light‐mantled sooty albatross and white‐chinned petrel) and carrion (northern and southern giant petrel; Figs 2a and 3). Although most previous community studies using conventional methods (Table 2) have not quantified fully the variation between and within foraging guilds, the descriptions of diet during the breeding period facilitate a comparison of patterns of feeding segregation in relation to the intrinsic characteristics of different oceanic environments. Temperate and polar communities foraging in productive ecosystems (i.e. frontal zones, coastal upwelling, highly productive shelves) typically include a wide diversity of feeding strategies including surface‐seizing, filtering, plunge and pursuit diving. Such communities, including that at South Georgia described in this study, include specialist planktivorous and piscivorous species, together with squid consumers, apex predator–scavengers and generalists that eat squid, fish and crustaceans in various proportions (Hobson, Piatt & Pitocchelli 1994; Ridoux 1994; Sydeman *et al*. 1997; this study). In contrast, tropical seabird communities feeding on marine environments that show limited seasonality and low productivity mainly consist of surface predators foraging in multispecies flocks that prey largely upon flying fish (family Exocoetidae) and squid (Diamond 1983; Harrison, Hida & Seki 1983).

**Table 2:** Published studies of seabird community structure based on conventional techniques or stable isotope analysis (SIA)

	Habitat type and location	No. of seabird species	Methods	Source
*Polar*	Iceland (north Atlantic)	6	Conventional	Lilliendahl & Solmundsson (1997)
	Iceland (north Atlantic)	6	SIA	Thompson *et al*. (1999)
	Svalbard region (northern Barents Sea)	6	Conventional	Mehlum & Gabrielsen (1993)
	Gulf of Alaska (north Pacific Ocean)	19	Conventional	Sanger (1987)
	Gulf of Alaska (north Pacific Ocean)	22	SIA	Hobson, Piatt & Pitocchelli (1994)
	Falkand Islands (south Atlantic Ocean)	8	Conventional vs. SIA	Weiss *et al*. (2009)
	Bird Island (south Atlantic Ocean)	8	SIA	Bodey *et al*. (2014)
	Macquarie Island (south Pacific Ocean)	9	Conventional	Goldsworthy *et al*. (2001)
	King George Island (Southern Ocean)	5	SIA	Quillfeldt, McGill & Furness (2005)
	Weddell Sea (Southern Ocean)	12	Conventional	Ainley *et al*. (1991)
	Weddell Sea (Southern Ocean)	12	Conventional vs. SIA	Rau *et al*. (1992)
*Tropical*	Subtropical Convergence (south Atlantic Ocean)	14	SIA	Bugoni, McGill & Furness (2010)
	Gulf of Farallones (north Pacific Ocean)	16	Conventional vs. SIA	Sydeman *et al*. (1997)
	Hawaiian Islands (north Pacific Ocean)	5	SIA	Bond *et al*. (2010)
	Line Island (central Pacific Ocean)	9	SIA	Young *et al*. (2010)
	Seychelles (west Indian Ocean)	8	Conventional	Catry *et al*. (2009)
	Christmas Island (south Pacific Ocean)	8	Conventional	Ashmole & Ashmole (1967)
	Europa Island (west Indian Ocean)	5	Conventional vs. SIA	Cherel *et al*. (2008)
*Temperate*	Chubut coast (south Atlantic Ocean)	15	SIA	Forero *et al*. (2004)

The comprehensive analysis reported here highlights the key food resources for the seabird community at South Georgia, which is in a region clearly affected by rapid and ongoing environmental change. The ecological significance of Antarctic krill for top predators in the Scotia Sea, including several of the albatrosses and petrels at South Georgia, has been pointed out previously (Croxall & Prince 1987; Croxall, Prince & Reid 1997; Murphy *et al*. 2007; Stowasser *et al*. 2012). Accordingly, when we considered all years and species, 32% of the community diet during the breeding period consisted of krill, supporting its prominent role in the transfer of energy from primary producers to seabirds. However, when considering all species except scavengers (northern and southern giant petrels), our analysis also highlighted that only 15 of the total of 120 prey species that were included (i.e. 12·5%) explained 75% of the differences between trophic guilds and comprised 78% of their diets. Thus, the structure of this community depended largely on the relative importance of a few key species of fish, crustaceans and squid, besides Antarctic krill (Table 3). This raises the issue that long‐term fluctuations in the availability of a small minority of prey, not just krill, will greatly increase interspecific competition.

**Table 3:** Percentages of wet mass for the most important 15 prey species in the diet of the procellariiform community breeding at Bird Island from 1974 to 2002 (Table 1)

	Wandering albatross	Black‐browed albatross	Grey‐headed albatross	Light‐mantled sooty albatross	Antarctic prion	Fairy Prion	Blue petrel	Common diving petrel	South Georgia diving petrel	White‐chinned petrel	Wilson's storm petrel	Northern giant petrel	Southern giant petrel
Squid													
*Kondakovia longimana*	22·0	7·2	12·9	0·9	0	0	0	0	0	2·5	0	5·9	1·0
*Moroteuthis knipovitchi*	2·6	2·0	2·3	0	0	0	0	0	0	0	0	0	0
*Martialia hyadesi*	0·6	12·8	27·5	0	0	0	0	0	0	2·8	0	0·1	0·2
*Todarodes sagittatus*	0·4	2·0	5·8	0	0	0	0·4	0	0	0	0	0	0
*Galiteuthis glacialis*	0·8	3·5	4·9	0	0	0	0	0	0	1·9	0	0·1	0·2
Fish													
*Dissostichus eleginoides*	28·0	0	0	0	0	0	0	0	0	0	0	1·6	1·0
*Champsocephalus gunnari*	1·0	13·5	5·7	0	0	0	0	0	0	3·1	0	0	0
*Pseudochaenichthys georgianus*	13·8	8·3	3·4	0	0	0	0	0	0	0	0	0	0
*Geotria australis*	0	0·1	8·3	0	0	0	0	0	0	0	0	0	0
*Gymnoscopelus nicholsi*	0	1·5	0·8	2·4	0	0	0	0	0	8·0	0	0	0
*Magnisudis prionosa*	0	6·3	6·3	0	0	0	0	0	0	0	0	0	0
Crustaceans													
*Euphausia superba*	0	37·4	18·5	38·4	41·7	80·1	74·6	13·4	59·4	47·4	36·1	18·1	16·0
*Calanoides acutus*	0	0	0	0	12·3	0·9	0·6	34·1	19·6	0	0	0	0
*Rhincalanus gigas*	0	0	0	0	21·6	3·2	0·7	25·3	4·3	0	0	0	0
*Themisto gaudichaudii*	0	0	0	0	4·8	15·8	0	4·8	0·2	0·7	31·2	0	0
Squid	26·4	27·4	53·4	0·9	0	0	0·4	0	0	7·2	0	6·0	1·4
Fish	42·7	29·7	24·6	2·4	0	0	0	0	0	11·1	0	1·6	1·0
Crustaceans	0	37·4	18·5	38·4	80·5	100·0	75·9	77·5	83·5	48·1	67·3	18·1	16·0
Combined prey	69·1	94·5	96·4	41·7	80·5	100·0	76·4	77·5	83·5	66·4	67·3	25·7	18·4

The 15 prey species are further aggregated into four major groups: squid, fish, crustaceans and all prey combined (bottom of table).

When feeding resources become less abundant, diet overlap may decrease and niche width increase because of the greater reliance on a wide range of suboptimal prey types (MacArthur & Pianka 1966; Krebs & Davies 1981). One compensatory response to low availability of a key prey is to switch to alternatives, which has been investigated at South Georgia only for white‐chinned petrels, grey‐headed and black‐browed albatrosses. Krill abundance was high throughout 1996 (*c. *26·7 g m^−^2) but apparently low in early 1998 (*c. *5 g m^−2^), yet the diet of white‐chinned petrels was similar between years, and krill was always the most important prey item followed by fish and squid (Berrow & Croxall 1999). In contrast, Croxall, Reid & Prince (1999) demonstrated that a fourfold difference in krill biomass between 1986 (*c. *30 g m^−2^) and 1997 (*c. *7 g m^−2^) around northwest South Georgia (Brierley, Watkins & Murray 1997) caused a reduction of 88–90% in the consumption of krill, and a compensatory increase of fish in the diet of both black‐browed and grey‐headed albatrosses (the latter also showed an increase in diet diversity). Although dietary overlap indices between the 2 years were very similar for the two albatrosses, the overlaps between albatrosses and penguins were greatly reduced in 1997.

An analysis of krill density in the Southern Ocean from 1926 to 2003 suggested a major decline since the 1970s as a consequence of the reduction in sea‐ice extent and duration (Atkinson *et al*. 2004; but see Loeba & Santorab 2015; Steinberg *et al*. 2015). Effects of a possible krill shortage on the guild structure of the procellariiform community remain untested. Despite the limitations associated with pooling of somewhat disparate data sets in this study, the analysis of conventional data for six species from three trophic guilds in multiple years has provided the first insights into possible temporal changes in the fundamental structure of the community at South Georgia. Although, as described above, variation between years in niche segregation may be influenced by abundance or availability of key prey, including krill, the temporal perspective provided here shows that, overall, the similarities remained much greater within than between the three trophic guilds, and thus, the main structure remains consistent through time (Fig. 4a).

The painstaking work involved in sorting and identifying diet samples to species is such that community‐level analyses are labour‐intensive, time‐consuming and therefore expensive. One potential means of overcoming this problem is to exploit the redundancy in community data by only analysing the samples to higher taxonomic levels, such as family. For the marine macro‐ and meio‐benthos, aggregations of species data to higher taxonomic levels have been used to assess how much information is lost compared with the full species‐level analysis (Olsgard, Somerfield & Carr 1998; Olsgard & Somerfield 2000). However, to our knowledge, there are no equivalent studies for seabird communities. The MDS and pairwise comparisons in our analyses confirm that the six species were grouped according to diet composition into the same three trophic guilds regardless of whether the analysis was carried out at species or family level (Fig. 4a,b). Therefore, our results demonstrate that a less intensive monitoring programme that involves prey identification only to a coarse taxonomic level can nevertheless provide reliable insights into the structure of seabird communities.

Although general community structure could be determined from aggregated data, it remained sensitive to taxonomic resolution. In particular, the similarity within and between species in different trophic guilds depended on the level to which prey were identified (Fig. 4). To illustrate, unusual oceanographic conditions in 2000 resulted in a much greater consumption of crustaceans by both grey‐headed and black‐browed albatrosses, and reduced reliance on what would otherwise have been their main prey, cephalopods and fish, respectively (Xavier, Croxall & Reid 2003). This switch was reflected in the analysis carried out at species and family level, which grouped together the diet of grey‐headed and black‐browed albatross in 2000 (Fig. 4a,b). However, some counter‐intuitive results arose as artefacts of analyses at coarser taxonomic levels (Fig. 4c). Therefore, low taxonomic resolution appears neither to be sufficient for detecting changes in the general community structure, nor meaningful ecological interpretation.

Biogeochemical markers such as δ^15^N reflect trophic level and have provided substantial insights into feeding ecology in previous studies of seabirds (Phillips *et al*. 2007; Moreno *et al*. 2010, 2013; Votier *et al*. 2010). However, in marine ecosystems, δ^15^N not only reflects trophic interactions but also correlates with nutrient availability and primary productivity (Graham *et al*. 2010; Stowasser *et al*. 2012). As a consequence of the simultaneous influence of diet and geographic variation in δ^15^N baselines, a difference between δ^15^N of consumers could indicate mainly a change in the inorganic nitrogen source utilized by primary producers, a different trophic position or a combination thereof. Information on isotopic ratios of potential prey from different foraging areas is critical for distinguishing the relative importance of prey vs. habitat specialization (Bugoni, McGill & Furness 2010; Moreno *et al*. 2011), particularly in regions where there are strong isotopic gradients. However, as these factors are usually to some extent conflated, especially in southern marine ecosystems, inferring seabird community structure from δ^15^N remains a challenge. Similarly, although some recent studies have demonstrated the potential of SIA for assessing the structure of large seabird communities at a scale of 1000–2000 km (Hobson, Piatt & Pitocchelli 1994; Forero *et al*. 2004; Bugoni, McGill & Furness 2010), these did not assess the influence of spatial variability on isotopic signatures of the various prey species.

The need to return regularly to provision chicks constrains the foraging range of breeding seabirds and therefore provides the opportunity for separating the effect of prey specialization from that of geographic variation in δ^15^N. The most detailed analysis to date of the food web within the foraging areas of the albatrosses and petrels included in our study (Stowasser *et al*. 2012) revealed a clear spatial variation in δ^15^N of particulate organic matter and several organisms, highlighting the wide intraspecific variability in isotopic signatures. A more targeted analysis restricted to potential prey of the procellariiform community indicated no significant differences between the δ^15^N of squid, fish and carrion, but a clear distinction between the δ^15^N of crustaceans and other types of prey (Fig. 6). Although only one species of squid was included in this analysis and there was no detectable difference in isotope ratios between squid, fish and carrion, a more complete study of δ^15^N in muscle of a wider range of squid species (Anderson *et al*. 2009) indicates that some have much higher δ^15^N than the species reported here (i.e. 10·51–11·36‰). Reflecting the differences in δ^15^N of potential prey (crustaceans vs. squid vs. fish and carrion), we found a clear correspondence between the four‐guild community structure obtained using conventional dietary data (Fig. 2a) and that using δ^15^N of chick feathers (Fig. 2b). The latter also discriminated species that feed mainly on crustaceans (Antarctic prion, blue petrel, common diving petrel, South Georgia diving petrel), large fish and squid (wandering albatross) and a mixture of crustaceans, small fish and squid (black‐browed albatross, grey‐headed albatross, light‐mantled sooty albatross, white‐chinned petrel). However, given the similarity in isotope ratios of fish and carrion (Fig. 6), analysis of δ^15^N of feathers failed to discriminate the scavenging giant petrels from black‐browed, grey‐headed and light‐mantled sooty albatrosses, and white‐chinned petrel. There were no data available for a direct assessment of temporal variation in the δ^15^N baseline across the very large foraging ranges of the procellariiform species included here. However, stable isotope ratios in chick feathers sampled from multiple species from 2001 to 2013 indicated that differences in δ^15^N between years were much less than those between trophic levels (Figs 5 and 6); hence, annual variation in baselines will have minimal impact on the isotopic assessment of trophic relationships. Although conventional dietary analysis provided better resolution of the community structure, our study also demonstrates that despite the potentially confounding influence of natural biogeochemical gradients in baseline stable isotope signature, δ^15^N in chick feathers is determined largely by trophic (level) specialization and therefore can also be used to monitor changes in the structure of the community.

Previous isotopic studies have highlighted that patchy knowledge of spatial heterogeneity in stable isotope signatures means that values in predator tissues require careful interpretations (Cherel & Hobson 2007; Phillips *et al*. 2009; Weiss *et al*. 2009; Moreno *et al*. 2011). The detailed picture of the seabird community and associated food web in the Antarctic ecosystem provided here indicates that to obtain a reliable estimate of trophic level or given the extensive overlap in isotope ratios, the proportion of a specific fish, squid or crustacean in the diet using mixing models may be impossible when diets are diverse, and seabirds feed in more than one water mass. As shown here, however, it is possible to use this pragmatic approach to reconstruct overall community trophic structure if the diets consist of components that are isotopically distinct at a coarse taxonomic level (crustaceans vs. fish and carrion vs. squid), providing an effective means for assessing long‐term changes in community interactions.

Our review highlighted that in the past four decades, barely 20 published studies have attempted to describe seabird communities, only seven of which considered more than 10 species and none monitored temporal changes (Table 2). By comparing conventional diet with isotopic data from predators, our analyses both explored the limitations and demonstrated the potential of combining multiple lines of evidence. The scarcity of such studies reflects a profound gap in knowledge of the basic mechanisms driving seabird community structure, and highlights the necessity of further research.

## References

[jane12434-bib-0001] Ainley, D.G. , Fraser, W.R. , Smith, W.O. , Hopkins, T.L. & Torres, J.J. (1991) The structure of upper level pelagic food webs in the Antarctic: effect of phytoplankton prey distribution. Journal of Marine Systems, 2, 111–122.

[jane12434-bib-0002] Anderson, O.R.J. , Phillips, R.A. , McDonald, R.A. , Shore, R.F. , McGill, R.A.R. & Bearhop, S. (2009) Influence of trophic position and foraging range on mercury levels within a seabird community. Marine Ecology Progress Series, 375, 277–288.

[jane12434-bib-0003] Ashmole, N.P. & Ashmole, M.J. (1967) Comparative feeding ecology of sea birds of a tropical oceanic island. Peabody Museum of Natural History and Department of Biology, Yale University Bulletin, 24, 1–128.

[jane12434-bib-0004] Atkinson, A. , Whitehouse, M.J. , Priddle, J. , Cripps, G.C. , Ward, P. & Brandon, M.A. (2001) South Georgia, Antarctica: a productive, cold water, pelagic ecosystem. Marine Ecology Progress Series, 216, 279–308.

[jane12434-bib-0005] Atkinson, A. , Siegel, V. , Pakhomov, E. & Rothery, P. (2004) Long‐term decline in krill stock and increase in salps within the Southern Ocean. Nature, 432, 100–103. 15525989 10.1038/nature02996

[jane12434-bib-0006] Beaugrand, G. , Luczak, C. & Edwards, M. (2009) Rapid biogeographical plankton shifts in the North Atlantic Ocean. Global Change Biology, 15, 1790–1803.

[jane12434-bib-0007] Berrow, S.D. & Croxall, J.P. (1999) The diet of white‐chinned petrels *Procellaria aequinoctialis,* Linnaeus 1758, in years of contrasting prey availability at South Georgia. Antarctic Science, 11, 283–292.

[jane12434-bib-0008] Bodey, T.W. , Ward, E.J. , Phillips, R.A. , McGill, R.A.R. & Bearhop, S. (2014) Species versus guild level differentiation revealed across the annual cycle by isotopic niche examination. Journal of Animal Ecology, 83, 470–478. 24215391 10.1111/1365-2656.12156

[jane12434-bib-0009] Bond, A.L. , McClelland, G.T.W. , Jones, I.L. , Lavers, J.L. & Kyser, T.K. (2010) Stable isotopes confirm community patterns in foraging among Hawaiian procellariiformes. Waterbirds, 33, 50–58.

[jane12434-bib-0010] Brierley, A.S. , Watkins, J.L. & Murray, A.W.A. (1997) lnterannual variability in krill abundance at South Georgia. Marine Ecology Progress Series, 150, 87–98.

[jane12434-bib-0011] Brown, C.J. , Fulton, E.A. , Hobday, A.J. , Matear, R.J. , Possingham, H.P. , Bulman, C. *et al* (2010) Effects of climate‐driven primary production change on marine food webs: implications for fisheries and conservation. Global Change Biology, 16, 1194–1212.

[jane12434-bib-0012] Bugoni, L. , McGill, R.A.R. & Furness, R.W. (2010) The importance of pelagic longline fishery discards for a seabird community determined through stable isotope analysis. Journal of Experimental Marine Biology and Ecology, 391, 190–200.

[jane12434-bib-0013] Carmel, Y. , Kent, R. , Bar‐Massada, A. , Blank, L. , Liberzon, J. , Nezer, O. *et al* (2013) Trends in ecological research during the last three decades – a systematic review. PLoS One, 8, e59813. 23637740 10.1371/journal.pone.0059813 PMC3634786

[jane12434-bib-0014] Catry, T. , Ramos, J.A. , Jaquemet, S. , Faulquier, L. , Berlincourt, M. , Hauselmann, A. *et al* (2009) Comparative foraging ecology of a tropical seabird community of the Seychelles, western Indian Ocean. Marine Ecology Progress Series, 374, 259–272.

[jane12434-bib-0015] Cherel, Y. & Hobson, K.A. (2007) Geographical variation in carbon stable isotope signatures of marine predators: a tool to investigate their foraging areas in the Southern Ocean. Marine Ecology Progress Series, 329, 281–287.

[jane12434-bib-0016] Cherel, Y. , Hobson, K.A. , Guinet, C. & Vanpe, C. (2007) Stable isotopes document seasonal changes in trophic niches and winter foraging individual specialization in diving predators from the Southern Ocean. Journal of Animal Ecology, 76, 826–836. 17584388 10.1111/j.1365-2656.2007.01238.x

[jane12434-bib-0017] Cherel, Y. , Corre, M.L. , Jaquemet, S. , Ménard, F. , Richard, P. & Weimerskirch, H. (2008) Resource partitioning within a tropical seabird community: new information from stable isotopes. Marine Ecology Progress Series, 366, 281–291.

[jane12434-bib-0100] Clarke, K.R. & Gorley, R.N. (2006) PRIMER version 6: User Manual/Tutorial. PRIMER‐E, Plymouth, UK, p. 192.

[jane12434-bib-0018] Clarke, K.R. , Somerfield, P.J. & Chapman, M.G. (2006) On resemblance measures for ecological studies, including taxonomic dissimilarities and a zero‐adjusted Bray–Curtis coefficient for denuded assemblages. Journal of Experimental Marine Biology and Ecology, 300, 55–80.

[jane12434-bib-0019] Clarke, A. , Croxall, J.P. , Poncet, S. , Martin, A.R. & Burton, R. (2012) Important bird areas: South Georgia. British Birds, 105, 118–144.

[jane12434-bib-0020] Croll, D.A. , Maron, J.L. , Estes, J.A. , Danner, E.M. & Byrd, G.V. (2005) Introduced Predators Transform Subarctic Islands from Grassland to Tundra. Science, 307, 1959–1961. 15790855 10.1126/science.1108485

[jane12434-bib-0021] Croxall, J.P. , North, A.W. & Prince, P.A. (1988) Fish prey of the wandering albatross *Diomedea exulans* at South Georgia. Polar Biology, 9, 9–16.

[jane12434-bib-0022] Croxall, J.P. & Prince, P.A. (1987) Seabirds as predators on marine resources, especially krill, at South Gerogia Seabirds: Feeding Biology and Role in Marine Ecosystems (ed. Croxall J.P.), pp. 347–367. Cambridge University Press, Cambridge, UK.

[jane12434-bib-0023] Croxall, J.P. , Prince, P.A. & Reid, K. (1997) Dietary segregation of krill‐eating South Georgia seabirds. Journal of Zoology, 242, 531–556.

[jane12434-bib-0024] Croxall, J.P. , Reid, K. & Prince, P.A. (1999) Diet, provisioning and productivity responses of marine predators to differences in availability of Antarctic krill. Marine Ecology Progress Series, 177, 115–131.

[jane12434-bib-0025] Croxall, J.P. , Trathan, P.N. & Murphy, E.J. (2002) Environmental change and Antarctic seabird populations. Science, 297, 1510–1514. 12202819 10.1126/science.1071987

[jane12434-bib-0026] Cury, P.M. , Boyd, I.L. , Bonhommeau, S. , Anker‐Nilssen, T. , Crawford, R.J.M. , Furness, R.W. *et al* (2011) Global seabird response to forage fish depletion: one‐third for the birds. Science, 334, 1703–1706. 22194577 10.1126/science.1212928

[jane12434-bib-0027] Diamond, A.W. (1983) Feeding overlap in some tropical and temperate seabird communities. Studies in Avian Biology, 8, 24–46.

[jane12434-bib-0028] Einoder, L.D. (2009) A review of the use of seabirds as indicators in fisheries and ecosystem management. Fisheries Research, 95, 6–13.

[jane12434-bib-0029] Forcada, J. & Trathan, P. (2009) Penguin responses to climate change in the Southern Ocean. Global Change Biology, 15, 1618–1630.

[jane12434-bib-0030] Forero, M.G. , Bortolotti, G.R. , Hobson, K.A. , Donazar, J.A. , Bertelloti, M. & Blanco, G. (2004) High trophic overlap within the seabird community of Argentinean Patagonia: a multiscale approach. Journal of Animal Ecology, 73, 789–801.

[jane12434-bib-0031] Frederiksen, M. , Edwards, M. , Richardson, A.J. , Halliday, N.C. & Wanless, S. (2006) From plankton to top predators: bottom‐up control of a marine food web across four trophic levels. Journal of Animal Ecology, 75, 1259–1268. 17032358 10.1111/j.1365-2656.2006.01148.x

[jane12434-bib-0032] Gille, S.T. (2002) Warming of the Southern Ocean since the 1950s. Science, 295, 1274–1277. 10.1126/science.1065863 11847337

[jane12434-bib-0033] Goldsworthy, S.D. , He, X. , Tuck, G.N. , Lewis, M. & Williams, R. (2001) Trophic interactions between the Patagonian toothfish, its fishery, and seals and seabirds around Macquarie Island. Marine Ecology Progress Series, 218, 283–302.

[jane12434-bib-0034] Graham, B.S. , Koch, P.L. , Newsome, S.D. , McMahon, K.W. & Aurioles, D. (2010) Using isoscapes to trace the movements and foraging behaviour of top predators in oceanic ecosystems Isoscapes. Understanding Movements, Pattern, and Process on Earth Through Isotope Mapping (eds West J.B., Bowen G.J., Dawson T.E. & Tu K.P.), pp. 299–318. Springer, New York, NY, USA.

[jane12434-bib-0035] Grandgeorge, M. , Wanless, S. , Dunn, T.E. , Maumy, M. , Beaugrand, G. & Gremillet, D. (2008) Resilience of the British and Irish seabird community in the twentieth century. Aquatic Biology, 4, 187–199.

[jane12434-bib-0036] Grémillet, D. & Charmantier, A. (2010) Shifts in phenotypic plasticity constrain the value of seabirds as ecological indicators of marine ecosystems. Ecological Applications, 20, 1498–1503. 20945754 10.1890/09-1586.1

[jane12434-bib-0037] Halpern, B.S. , Walbridge, S. , Selkoe, K.A. , Kappel, C.V. , Micheli, F. , D'Agrosa, C. *et al* (2008) A global map of human impact on marine ecosystems. Science, 319, 948–952. 18276889 10.1126/science.1149345

[jane12434-bib-0038] Harrison, C.S. , Hida, T.S. & Seki, M.P. (1983) Hawaiian seabird feeding ecology. Wildlife Monographs, 85, 1–71.

[jane12434-bib-0039] Hill, S.L. , Phillips, T. & Atkinson, A. (2013) Potential Climate Change Effects on the Habitat of Antarctic Krill in the Weddell Quadrant of the Southern Ocean. PLoS One, 8, 72246. 10.1371/journal.pone.0072246 PMC3749108 23991072

[jane12434-bib-0040] Hobson, K.A. , Piatt, J.F. & Pitocchelli, J. (1994) Using stable isotopes to determine seabird trophic relationships. Journal of Animal Ecology, 63, 786–798.

[jane12434-bib-0041] Hoegh‐Guldberg, O. & Bruno, J.F. (2010) The impact of climate change on the world's marine ecosystems. Science, 328, 1523–1528. 20558709 10.1126/science.1189930

[jane12434-bib-0042] Hunter, S. (1983) The food and feeding of the giant petrels *Macronectes halli* and *M.giganteus* at South Georgia. Journal of Zoology, London, 200, 521–538.

[jane12434-bib-0043] Karnovsky, N.J. , Hobson, K.J. & Iverson, S.J. (2012) From lavage to lipids: innovations and limitations in estimating diets of seabirds. Marine Ecology Progress Series, 451, 263–284.

[jane12434-bib-0044] Krebs, J.R. & Davies, N.B. (1981) An Introduction to Behavioural Ecology. Blackwell, Oxford, UK.

[jane12434-bib-0045] Lauria, V. , Attrill, M.J. , Brown, A. , Edwards, M. & Votier, S.C. (2013) Regional variation in the impact of climate change: evidence that bottom‐up regulation from plankton to seabirds is weak in parts of the Northeast Atlantic. Marine Ecology Progress Series, 488, 11–22.

[jane12434-bib-0046] Layman, C.A. , Araujo, M.S. , Boucek, R. , Hammerschlag‐Peyer, C.M. , Harrison, E. , Jud, Z.R. *et al* (2012) Applying stable isotopes to examine food‐web structures: an overview of analytical tools. Biological Reviews, 87, 545–562. 22051097 10.1111/j.1469-185X.2011.00208.x

[jane12434-bib-0047] Levitus, S. , Antonov, J.I. , Boyer, T.P. & Stephens, C. (2000) Warming of the world ocean. Science, 287, 2225–2228.

[jane12434-bib-0048] Lewison, R. , Oro, D. , Godley, B.J. , Underhill, L. , Bearhop, S. , Wilson, R.P. *et al* (2012) Research priorities for seabirds: improving conservation and management in the 21st century. Endangered Species Research, 17, 93–121.

[jane12434-bib-0049] Liddle, G.M. (1994) Interannual variation in the breeding biology of the Antarctic prion *Pachyptila desolata* at Bird Island, South Georgia. Journal of Zoology, 234, 125–139.

[jane12434-bib-0050] Lilliendahl, K. & Solmundsson, J. (1997) An estimate of summer food consumption of six seabird species in Iceland. ICES Journal of Marine Science, 54, 624–630.

[jane12434-bib-0051] Link, J.S. & Garrison, L.P. (2002) Trophic ecology of Atlantic cod *Gadus morhua* on the northeast US continental shelf. Marine Ecology Progress Series, 227, 109–123.

[jane12434-bib-0052] Loeba, V.J. & Santorab, J.A. (2015) Climate variability and spatiotemporal dynamics of five Southern Ocean krill species. Progress in Oceanography, 134, 93–122.

[jane12434-bib-0053] MacArthur, R.H. & Pianka, E.R. (1966) On optimal use of patchy environment. The American Naturalist, 100, 603–609.

[jane12434-bib-0054] Mehlum, F. & Gabrielsen, W. (1993) The diet of high‐arctic seabirds in coastal and ice‐covered, pelagic areas near the Svalbard archipelago. Polar Research, 12, 1–20.

[jane12434-bib-0055] Ménard, F. , Lorrain, A. , Potier, M. & Marsac, F. (2007) Isotopic evidence of distinct foraging ecology and movement pattern in two migratory predators (yellowfin tuna and swordfish) of the western Indian Ocean. Marine Biology, 153, 141–152.

[jane12434-bib-0056] Miller, A.K. & Sydeman, W.J. (2004) Rockfish response to low‐frequency ocean climate change as revealed by the diet of a marine bird over multiple time scales. Marine Ecology Progress Series, 281, 207–216.

[jane12434-bib-0057] Montevecchi, W.A. (2007) Binary dietary responses of northern gannets *Sula bassana* indicate changing food web and oceanographic conditions. Marine Ecology Progress Series, 352, 213–220.

[jane12434-bib-0058] Moreno, R. , Jover, L. , Munilla, I. , Velando, A. & Sanpera, C. (2010) A three‐isotope approach to disentangling the diet of a generalist consumer: the yellow‐legged gull in northwest Spain. Marine Biology, 157, 545–553.

[jane12434-bib-0059] Moreno, R. , Jover, L. , Velando, A. , Munilla, I. & Sanpera, C. (2011) Influence of trophic ecology and spatial variation on the isotopic fingerprints of seabirds. Marine Ecology Progress Series, 442, 229–239.

[jane12434-bib-0060] Moreno, R. , Jover, L. , Diez, C. , Sardà‐Palomera, F. & Sanpera, C. (2013) Ten years after the prestige oil spill: seabird trophic ecology as indicator of long‐term effects on the coastal marine ecosystem. PLoS One, 8, e77360. 24130877 10.1371/journal.pone.0077360 PMC3793948

[jane12434-bib-0061] Murphy, E.J. , Watkins, J.L. , Trathan, P.N. , Reid, K. , Meredith, M.P. , Thorpe, S.E. *et al* (2007) Spatial and temporal operation of the Scotia Sea ecosystem: a review of large‐scale links in a krill centred food web. Philosophical Transactions of the Royal Society of London, Part B: Biological Sciences, 362, 113–148. 10.1098/rstb.2006.1957 PMC1764830 17405210

[jane12434-bib-0062] Olsgard, F. & Somerfield, P.J. (2000) Surrogates in marine benthic investigations – which taxonomic unit to target? Journal of Aquatic Ecosystem Stress and Recovery, 7, 25–42.

[jane12434-bib-0063] Olsgard, F. , Somerfield, P.J. & Carr, M.R. (1998) Relationships between taxonomic resolution, macrobenthic community patterns and disturbance. Marine Ecology Progress Series, 172, 25–36.

[jane12434-bib-0064] Payne, M.R. & Prince, P.A. (1979) Identification and breeding biology of the diving petrels *Pelecanoides georgicus* and *P. urinatrix exsul* at South Georgia. The New Zealand Journal of Zoology, 6, 299–318.

[jane12434-bib-0065] Phillips, R.A. , Catry, P. , Silk, J.R.D. , Bearhop, S. , McGill, R. , Afanasyev, V. *et al* (2007) Movements, winter distribution and activity patterns of Falkland and brown skuas: insights from loggers and isotopes. Marine Ecology Progress Series, 345, 281–291.

[jane12434-bib-0066] Phillips, R.A. , Bearhop, S. , Mcgill, R.A.R. & Dawson, D.A. (2009) Stable isotopes reveal individual variation in migration strategies and habitat preferences in a suite of seabirds during the nonbreeding period. Oecologia, 160, 795–806. 19377898 10.1007/s00442-009-1342-9

[jane12434-bib-0067] Piatt, J.F. , Sydeman, W.J. & Browman, H.I. (2007) Seabirds as indicators of marine ecosystems. Marine Ecology Progress Series, 352, 199–204.

[jane12434-bib-0068] Prince, P.A. (1979) The food and feeding ecology of grey‐headed albatross *Diomedea chrysostoma* and black‐browed albatross *D. melanophris* . Ibis, 122, 476–488.

[jane12434-bib-0069] Prince, P.A. (1980) The food and feeding ecology of Blue petrel (*Halobaena caerulea*) and Dove prion (*Pachyptila desolata*). Journal of Zoology, London, 190, 59–76.

[jane12434-bib-0070] Prince, P.A. & Copestake, P.G. (1990) Diet and aspects of fairy prions breeding at South Georgia. Notornis, 37, 59–69.

[jane12434-bib-0071] Quillfeldt, P. , McGill, R.A.R. & Furness, R.W. (2005) Diet and foraging areas of Southern Ocean seabirds and their prey inferred from stable isotopes: review and case study of Wilson's storm‐petrel. Marine Ecology Progress Series, 295, 295–304.

[jane12434-bib-0072] Rau, G.H. , Ainley, D.G. , Bengtson, J.L. , Torres, J.J. & Hopkins, T.L. (1992) ^15^N/^14^N and ^13^C/^12^C in Weddell Sea birds, seals, and fish: implications for diet and trophic structure. Marine Ecology Progress Series, 84, 1–8.

[jane12434-bib-0073] Reid, K. , Croxall, J.P. & Edwards, T.M. (1997a) Interannual variation in the diet of the Antarctic prion *Pachyptila desolata* at South Georgia. Emu, 97, 126–132.

[jane12434-bib-0074] Reid, K. , Croxall, J.P. , Edwards, T.M. , Hill, H.J. & Prince, P.A. (1997b) Diet and feeding ecology of the diving petrels *Pelecanoides georgicus* and *P. urinatrix* at South Georgia. Polar biology, 17, 17–24.

[jane12434-bib-0075] Ridoux, V. (1994) The diets and dietary segregation of seabirds at the subantarctic Crozet Islands. Marine Ornithology, 22, 1–192.

[jane12434-bib-0076] Rodhouse, P.G. , Clarke, M.R. & Murray, W.A. (1987) Cephalopod prey of the wandering albatross *Diomedea exulans* . Marine Biology, 96, 1–10.

[jane12434-bib-0077] Roscales, J.L. , Gómez‐Díaz, E. , Neves, V. & González‐Solís, J. (2011) Trophic versus geographic structure in stable isotope signatures of pelagic seabirds breeding in the northeast Atlantic. Marine Ecology Progress Series, 434, 1–13.

[jane12434-bib-0078] Sandvik, H. , Coulson, T. & Saether, B.E. (2008) A latitudinal gradient in climate effects on seabird demography: results from interspecific analyses. Global Change Biology, 14, 703–713.

[jane12434-bib-0079] Sanger, G.A. (1987) Trophic levels and trophic relationships of seabirds in the Gulf of Alaska Seabirds: Feeding Ecology and Role in Marine Ecosystems (ed. Croxall J.P.), pp. 229–257. Cambridge University Press, Cambridge, UK.

[jane12434-bib-0080] Smith, A.D.M. , Brown, C.J. , Bulman, C.M. , Fulton, E.A. , Johnson, P. , Kaplan, I.C. *et al* (2011) Impacts of fishing low‐trophic level species on marine ecosystems. Science, 333, 1147–1150. 21778363 10.1126/science.1209395

[jane12434-bib-0081] Somerfield, P.J. , Clarke, K.R. & Olsgard, F. (2002) A comparison of the power of categorical and correlational tests applied to community ecology data from gradient studies. Journal of Animal Ecology, 71, 581–593.

[jane12434-bib-0082] Steinberg, D.K. , Ruck, K.E. , Gleiber, M.R. , Garzio, L.M. , Cope, J.S. , Bernard, K.S. *et al* (2015) Long‐term (1993–2013) changes in macrozooplankton off the Western Antarctic Peninsula. Deep Sea Research Part I: Oceanographic Research Papers, 101, 54–70.

[jane12434-bib-0083] Stowasser, G. , Atkinson, A. , McGill, R.A.R. , Phillips, R.A. , Collins, M.A. & Pond, D.W. (2012) Food web dynamics in the Scotia Sea in summer: a stable isotope study. Deep‐Sea Research II: Topical Studies in Oceanography, 59–60, 208–221.

[jane12434-bib-0084] Sydeman, W.J. , Hobson, K.A. , Pyle, P. & McLaren, E.B. (1997) Trophic relationships among seabirds in central California: combined stable isotope and conventional dietary approach. The Condor, 99, 327–336.

[jane12434-bib-0085] Thomas, G. (1981) The food and feeding ecology of the light‐mantled sooty albatross at South Georgia. Emu, 82, 92–100.

[jane12434-bib-0086] Thompson, D.R. , Lilliendahl, K. , Solmundsson, J. , Furness, R.W. , Waldron, S. & Phillips, R.A. (1999) Trophic relationships among six species of Icelandic seabirds as determined through stable isotope analysis. The Condor, 101, 898–903.

[jane12434-bib-0087] Trathan, P.N. , Forcada, J. & Murphy, E.J. (2007) Environmental forcing and Southern Ocean marine predator populations: effects of climate change and variability. Philosophical Transactions of the Royal Society of London, Part B: Biological Sciences, 362, 2351–2365. 10.1098/rstb.2006.1953 PMC2443178 17553770

[jane12434-bib-0088] Trathan, P.N. & Reid, K. (2009) Exploitation of the marine ecosystem in the sub‐antarctic: historical impacts and current consequences. Papers and Proceedings of the Royal Society of Tasmania, 143, 9–14.

[jane12434-bib-0089] Votier, S.C. , Birkhead, T.R. , Oro, D. , Trinder, M. , Grantham, M.J. , Clark, J.A. *et al* (2008) Recruitment and survival of immature seabirds in relation to oil spills and climate variability. Journal of Animal Ecology, 77, 974–983. 18624739 10.1111/j.1365-2656.2008.01421.x

[jane12434-bib-0090] Votier, S.C. , Bearhop, S. , Witt, M.J. , Inger, R. , Thompson, D. & Newton, J. (2010) Individual responses of seabirds to commercial fisheries revealed using GPS tracking, stable isotopes and vessel monitoring systems. Journal of Applied Ecology, 47, 487–497.

[jane12434-bib-0091] Weiss, F. , Furness, R.W. , McGill, R.A.R. , Strange, I.J. , Masello, J.F. & Quillfeldt, P. (2009) Trophic segregation of Falkland Islands seabirds: insights from stable isotope analysis. Polar Biology, 32, 1753–1763.

[jane12434-bib-0092] Xavier, J.C. , Croxall, J.P. & Reid, K. (2003) Interannual variation in the diets of two albatross species breeding at South Georgia: implications for breeding performance. Ibis, 145, 593–610.

[jane12434-bib-0093] Young, H.S. , McCauley, D.J. , Dirzo, R. , Dunbar, R.B. & Shaffer, S.A. (2010) Niche partitioning among and within sympatric tropical seabirds revealed by stable isotope analysis. Marine Ecology Progress Series, 416, 285–294.

